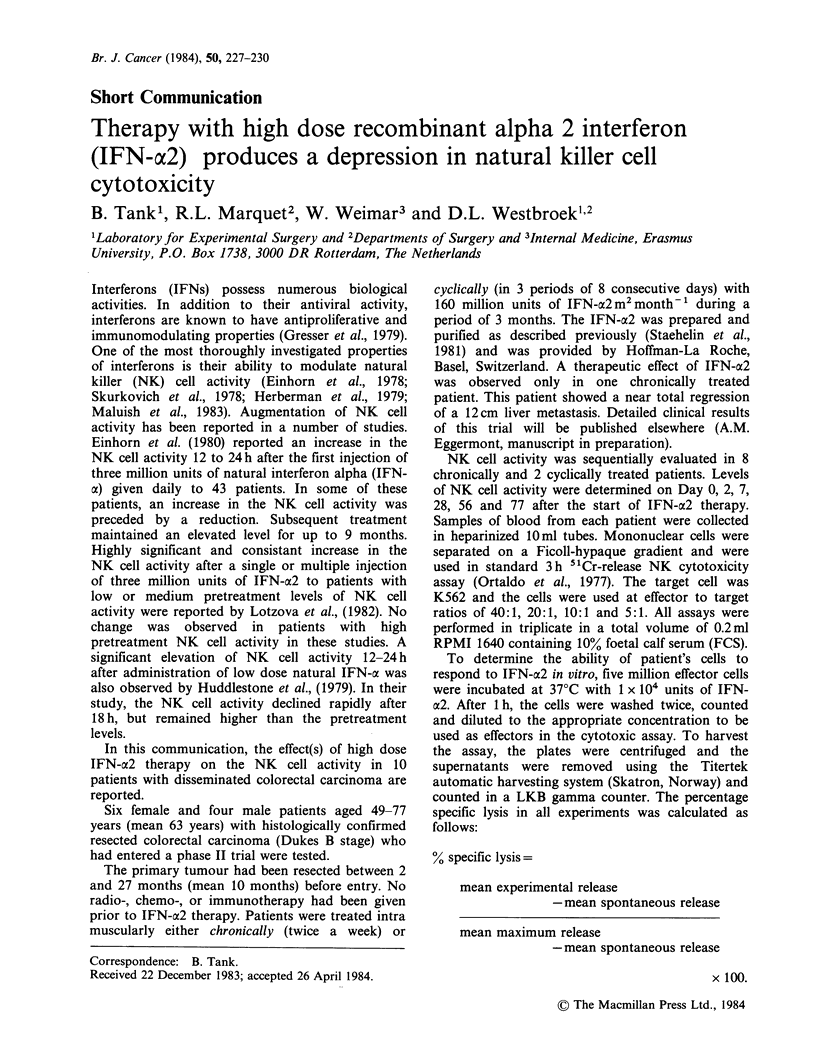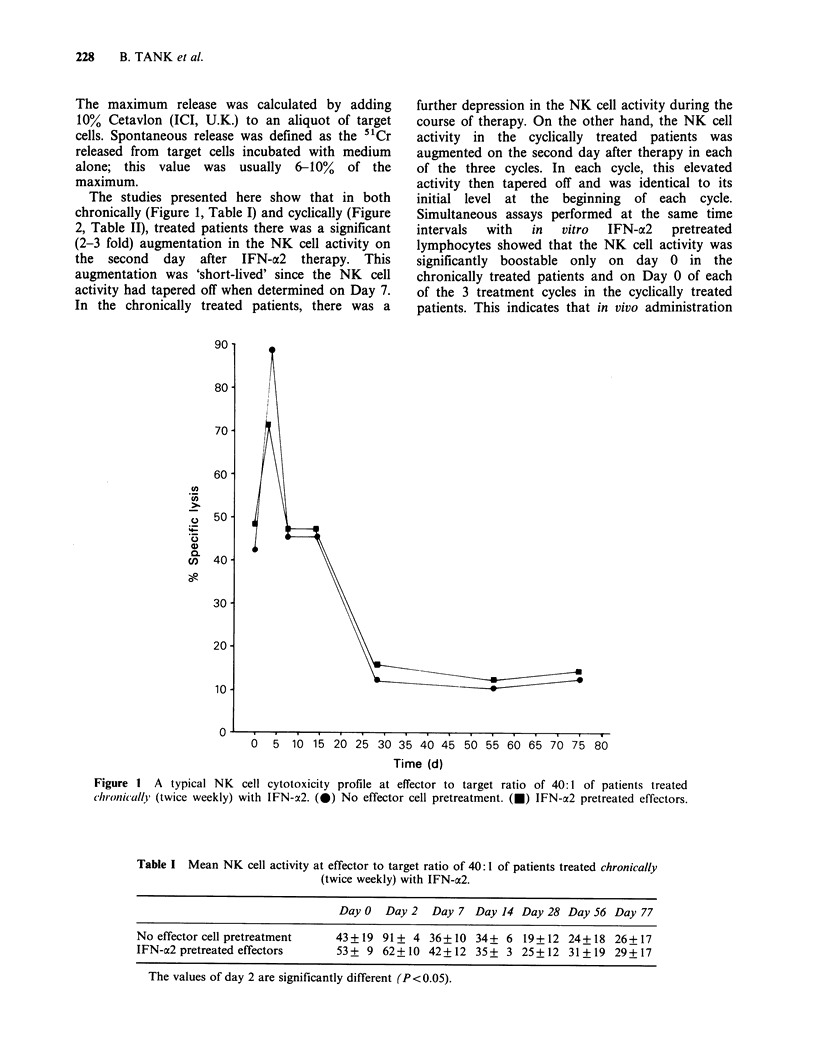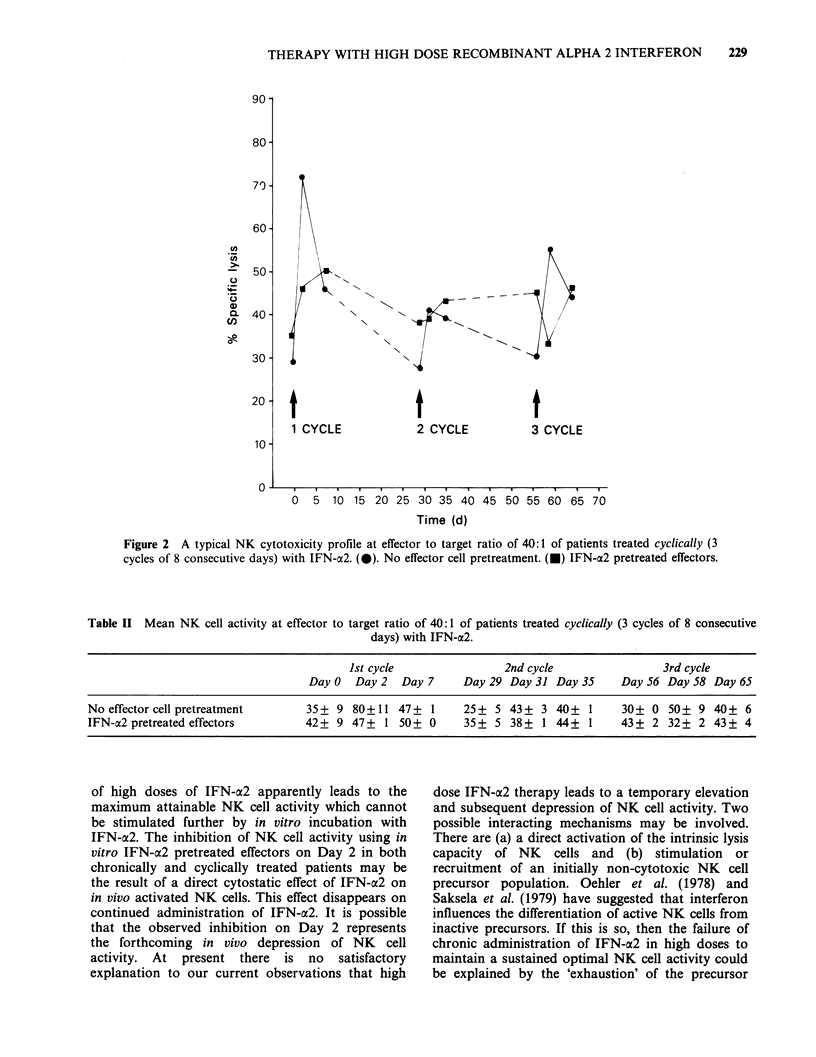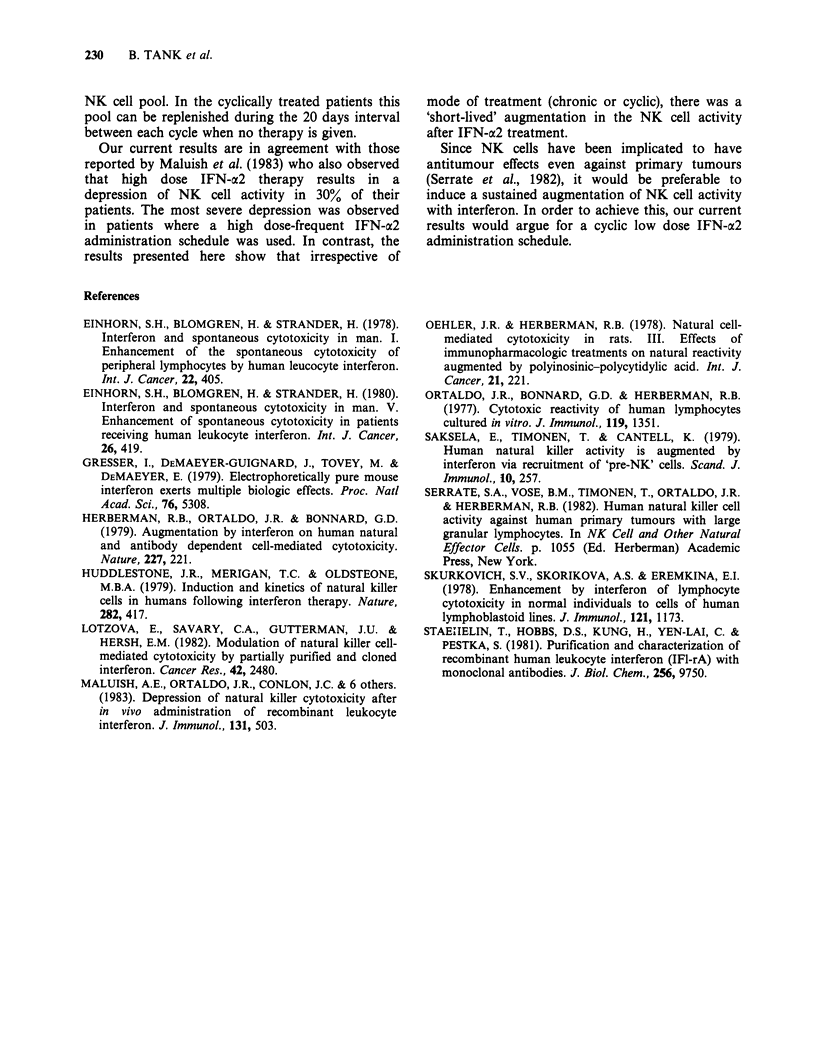# Therapy with high dose recombinant alpha 2 interferon (IFN-alpha 2) produces a depression in natural killer cell cytotoxicity.

**DOI:** 10.1038/bjc.1984.167

**Published:** 1984-08

**Authors:** B. Tank, R. L. Marquet, W. Weimar, D. L. Westbroek


*Br. J. Cancer* (1984), **50**, 227–230

**Short Communication**

### Therapy with high dose recombinant alpha 2 interferon (IFN-α2) produces a depression in natural killer cell cytotoxicity

B. Tank[1], R.L. Marquet[2], W. Weimar[3] and D.L. Westbroek[1,2]

[1]*Laboratory for Experimental Surgery and* [2]*Departments of Surgery and* [3]*Internal Medicine, Erasmus University, P.O. Box 1738, 3000 DR Rotterdam, The Netherlands*

Interferons (IFNs) possess numerous biological activities. In addition to their antiviral activity, interferons are known to have antiproliferative and immunomodulating properties (Gresser *et al.*, 1979). One of the most thoroughly investigated properties of interferons is their ability to modulate natural killer (NK) cell activity (Einhorn *et al.*, 1978; Skurkovich *et al.*, 1978; Herberman *et al.*, 1979; Maluish *et al.*, 1983). Augmentation of NK cell activity has been reported in a number of studies. Einhorn *et al.* (1980) reported an increase in the NK cell activity 12 to 24 h after the first injection of three million units of natural interferon alpha (IFN-α) given daily to 43 patients. In some of these patients, an increase in the NK cell activity was preceded by a reduction. Subsequent treatment maintained an elevated level for up to 9 months. Highly significant and consistant increase in the NK cell activity after a single or multiple injection of three million units of IFN-α2 to patients with low or medium pretreatment levels of NK cell activity were reported by Lotzova *et al.*, (1982). No change was observed in patients with high pretreatment NK cell activity in these studies. A significant elevation of NK cell activity 12–24 h after administration of low dose natural IFN-α was also observed by Huddlestone *et al.*, (1979). In their study, the NK cell activity declined rapidly after 18 h, but remained higher than the pretreatment levels.

In this communication, the effect(s) of high dose IFN-α2 therapy on the NK cell activity in 10 patients with disseminated colorectal carcinoma are reported.

Six female and four male patients aged 49–77 years (mean 63 years) with histologically confirmed resected colorectal carcinoma (Dukes B stage) who had entered a phase II trial were tested.

The primary tumour had been resected between 2 and 27 months (mean 10 months) before entry. No radio-, chemo-, or immunotherapy had been given prior to IFN-α2 therapy. Patients were treated intra muscularly either *chronically* (twice a week) or *cyclically* (in 3 periods of 8 consecutive days) with 160 million units of IFN-α2 $m^2$ $month^{-1}$ during a period of 3 months. The IFN-α2 was prepared and purified as described previously (Staehelin *et al.*, 1981) and was provided by Hoffman-La Roche, Basel, Switzerland. A therapeutic effect of IFN-α2 was observed only in one chronically treated patient. This patient showed a near total regression of a 12 cm liver metastasis. Detailed clinical results of this trial will be published elsewhere (A.M. Eggermont, manuscript in preparation).

NK cell activity was sequentially evaluated in 8 chronically and 2 cyclically treated patients. Levels of NK cell activity were determined on Day 0, 2, 7, 28, 56 and 77 after the start of IFN-α2 therapy. Samples of blood from each patient were collected in heparinized 10 ml tubes. Mononuclear cells were separated on a Ficoll-hypaque gradient and were used in standard 3 h $^{51}Cr$-release NK cytotoxicity assay (Ortaldo *et al.*, 1977). The target cell was K562 and the cells were used at effector to target ratios of 40:1, 20:1, 10:1 and 5:1. All assays were performed in triplicate in a total volume of 0.2 ml RPMI 1640 containing 10% foetal calf serum (FCS).

To determine the ability of patient's cells to respond to IFN-α2 *in vitro*, five million effector cells were incubated at 37°C with $1 \times 10^4$ units of IFN-α2. After 1 h, the cells were washed twice, counted and diluted to the appropriate concentration to be used as effectors in the cytotoxic assay. To harvest the assay, the plates were centrifuged and the supernatants were removed using the Titertek automatic harvesting system (Skatron, Norway) and counted in a LKB gamma counter. The percentage specific lysis in all experiments was calculated as follows:

$$\% \text{ specific lysis} = \frac{\text{mean experimental release} - \text{mean spontaneous release}}{\text{mean maximum release} - \text{mean spontaneous release}} \times 100.$$

Correspondence: B. Tank.
Received 22 December 1983; accepted 26 April 1984.

© The Macmillan Press Ltd., 1984

The maximum release was calculated by adding 10% Cetavlon (ICI, U.K.) to an aliquot of target cells. Spontaneous release was defined as the $^{51}Cr$ released from target cells incubated with medium alone; this value was usually 6–10% of the maximum.

The studies presented here show that in both chronically (Figure 1, Table I) and cyclically (Figure 2, Table II), treated patients there was a significant (2–3 fold) augmentation in the NK cell activity on the second day after IFN-α2 therapy. This augmentation was 'short-lived' since the NK cell activity had tapered off when determined on Day 7. In the chronically treated patients, there was a further depression in the NK cell activity during the course of therapy. On the other hand, the NK cell activity in the cyclically treated patients was augmented on the second day after therapy in each of the three cycles. In each cycle, this elevated activity then tapered off and was identical to its initial level at the beginning of each cycle. Simultaneous assays performed at the same time intervals with *in vitro* IFN-α2 pretreated lymphocytes showed that the NK cell activity was significantly boostable only on day 0 in the chronically treated patients and on Day 0 of each of the 3 treatment cycles in the cyclically treated patients. This indicates that *in vivo* administration

**Figure 1** A typical NK cell cytotoxicity profile at effector to target ratio of 40:1 of patients treated *chronically* (twice weekly) with IFN-α2. (●) No effector cell pretreatment. (■) IFN-α2 pretreated effectors.

**Table I** Mean NK cell activity at effector to target ratio of 40:1 of patients treated *chronically* (twice weekly) with IFN-α2.

| | *Day 0* | *Day 2* | *Day 7* | *Day 14* | *Day 28* | *Day 56* | *Day 77* |
|---|---|---|---|---|---|---|---|
| No effector cell pretreatment | 43±19 | 91± 4 | 36±10 | 34± 6 | 19±12 | 24±18 | 26±17 |
| IFN-α2 pretreated effectors | 53± 9 | 62±10 | 42±12 | 35± 3 | 25±12 | 31±19 | 29±17 |

The values of day 2 are significantly different ($P<0.05$).

**Figure 2** A typical NK cytotoxicity profile at effector to target ratio of 40:1 of patients treated *cyclically* (3 cycles of 8 consecutive days) with IFN-α2. (●). No effector cell pretreatment. (■) IFN-α2 pretreated effectors.

**Table II** Mean NK cell activity at effector to target ratio of 40:1 of patients treated *cyclically* (3 cycles of 8 consecutive days) with IFN-α2.

| | *1st cycle* | | | *2nd cycle* | | | *3rd cycle* | | |
|---|---|---|---|---|---|---|---|---|---|
| | *Day 0* | *Day 2* | *Day 7* | *Day 29* | *Day 31* | *Day 35* | *Day 56* | *Day 58* | *Day 65* |
| No effector cell pretreatment | 35± 9 | 80±11 | 47± 1 | 25± 5 | 43± 3 | 40± 1 | 30± 0 | 50± 9 | 40± 6 |
| IFN-α2 pretreated effectors | 42± 9 | 47± 1 | 50± 0 | 35± 5 | 38± 1 | 44± 1 | 43± 2 | 32± 2 | 43± 4 |

of high doses of IFN-α2 apparently leads to the maximum attainable NK cell activity which cannot be stimulated further by *in vitro* incubation with IFN-α2. The inhibition of NK cell activity using *in vitro* IFN-α2 pretreated effectors on Day 2 in both chronically and cyclically treated patients may be the result of a direct cytostatic effect of IFN-α2 on *in vivo* activated NK cells. This effect disappears on continued administration of IFN-α2. It is possible that the observed inhibition on Day 2 represents the forthcoming *in vivo* depression of NK cell activity. At present there is no satisfactory explanation to our current observations that high dose IFN-α2 therapy leads to a temporary elevation and subsequent depression of NK cell activity. Two possible interacting mechanisms may be involved. There are (a) a direct activation of the intrinsic lysis capacity of NK cells and (b) stimulation or recruitment of an initially non-cytotoxic NK cell precursor population. Oehler *et al.* (1978) and Saksela *et al.* (1979) have suggested that interferon influences the differentiation of active NK cells from inactive precursors. If this is so, then the failure of chronic administration of IFN-α2 in high doses to maintain a sustained optimal NK cell activity could be explained by the 'exhaustion' of the precursor

NK cell pool. In the cyclically treated patients this pool can be replenished during the 20 days interval between each cycle when no therapy is given.

Our current results are in agreement with those reported by Maluish *et al.* (1983) who also observed that high dose IFN-$\alpha$2 therapy results in a depression of NK cell activity in 30% of their patients. The most severe depression was observed in patients where a high dose-frequent IFN-$\alpha$2 administration schedule was used. In contrast, the results presented here show that irrespective of mode of treatment (chronic or cyclic), there was a 'short-lived' augmentation in the NK cell activity after IFN-$\alpha$2 treatment.

Since NK cells have been implicated to have antitumour effects even against primary tumours (Serrate *et al.*, 1982), it would be preferable to induce a sustained augmentation of NK cell activity with interferon. In order to achieve this, our current results would argue for a cyclic low dose IFN-$\alpha$2 administration schedule.

### References

EINHORN, S.H., BLOMGREN, H. & STRANDER, H. (1978). Interferon and spontaneous cytotoxicity in man. I. Enhancement of the spontaneous cytotoxicity of peripheral lymphocytes by human leucocyte interferon. *Int. J. Cancer*, **22**, 405.

EINHORN, S.H., BLOMGREN, H. & STRANDER, H. (1980). Interferon and spontaneous cytotoxicity in man. V. Enhancement of spontaneous cytotoxicity in patients receiving human leukocyte interferon. *Int. J. Cancer*, **26**, 419.

GRESSER, I., DEMAEYER-GUIGNARD, J., TOVEY, M. & DEMAEYER, E. (1979). Electrophoretically pure mouse interferon exerts multiple biologic effects. *Proc. Natl Acad. Sci.*, **76**, 5308.

HERBERMAN, R.B., ORTALDO, J.R. & BONNARD, G.D. (1979). Augmentation by interferon on human natural and antibody dependent cell-mediated cytotoxicity. *Nature*, **227**, 221.

HUDDLESTONE, J.R., MERIGAN, T.C. & OLDSTEONE, M.B.A. (1979). Induction and kinetics of natural killer cells in humans following interferon therapy. *Nature*, **282**, 417.

LOTZOVA, E., SAVARY, C.A., GUTTERMAN, J.U. & HERSH, E.M. (1982). Modulation of natural killer cell-mediated cytotoxicity by partially purified and cloned interferon. *Cancer Res.*, **42**, 2480.

MALUISH, A.E., ORTALDO, J.R., CONLON, J.C. & 6 others. (1983). Depression of natural killer cytotoxicity after *in vivo* administration of recombinant leukocyte interferon. *J. Immunol.*, **131**, 503.

OEHLER, J.R. & HERBERMAN, R.B. (1978). Natural cell-mediated cytotoxicity in rats. III. Effects of immunopharmacologic treatments on natural reactivity augmented by polyinosinic–polycytidylic acid. *Int. J. Cancer*, **21**, 221.

ORTALDO, J.R., BONNARD, G.D. & HERBERMAN, R.B. (1977). Cytotoxic reactivity of human lymphocytes cultured *in vitro*. *J. Immunol.*, **119**, 1351.

SAKSELA, E., TIMONEN, T. & CANTELL, K. (1979). Human natural killer activity is augmented by interferon via recruitment of 'pre-NK' cells. *Scand. J. Immunol.*, **10**, 257.

SERRATE, S.A., VOSE, B.M., TIMONEN, T., ORTALDO, J.R. & HERBERMAN, R.B. (1982). Human natural killer cell activity against human primary tumours with large granular lymphocytes. In *NK Cell and Other Natural Effector Cells*. p. 1055 (Ed. Herberman) Academic Press, New York.

SKURKOVICH, S.V., SKORIKOVA, A.S. & EREMKINA, E.I. (1978). Enhancement by interferon of lymphocyte cytotoxicity in normal individuals to cells of human lymphoblastoid lines. *J. Immunol.*, **121**, 1173.

STAEHELIN, T., HOBBS, D.S., KUNG, H., YEN-LAI, C. & PESTKA, S. (1981). Purification and characterization of recombinant human leukocyte interferon (IFl-rA) with monoclonal antibodies. *J. Biol. Chem.*, **256**, 9750.